# Anatomical and Technical Reappraisal of the Pallidothalamic Tractotomy With the Incisionless Transcranial MR-Guided Focused Ultrasound. A Technical Note

**DOI:** 10.3389/fsurg.2019.00002

**Published:** 2019-01-24

**Authors:** Marc N. Gallay, David Moser, Christian Federau, Daniel Jeanmonod

**Affiliations:** ^1^Center for Ultrasound Functional Neurosurgery, SoniModul, Solothurn, Switzerland; ^2^Institute for Biomedical Engineering, ETH Zürich, University of Zürich, Zurich, Switzerland; ^3^Department of Radiology, University Hospital Basel, Basel, Switzerland

**Keywords:** pallidothalamic tractotomy, functional stereotactic neurosurgery, minimal invasive, incisionless, cumulative equivalent minutes at 43°C, high intensity MR-guided focused ultrasound, subthalamotomy, Parkinson's disease

## Abstract

**Background:** MR-guided focused ultrasound (MRgFUS) offers new perspectives for safe and efficient lesioning inside the brain. The issue of target coverage remains primordial and sub-optimally addressed or solved in the field of functional neurosurgery.

**Objective:** To provide an optimized planning and operative strategy to perform a pallidothalamic tractotomy (PTT) in chronic therapy-resistant Parkinson's disease (PD) with the technology of MRgFUS.

**Methods and results:** Histological sections and maps from 6 human brain hemispheres were analyzed and outlines of the pallidothalamic tract on Myelin-stained sections were drawn and superimposed. We determined a standardized PTT target coverage characterized by 5 to 7 preplanned target lesion sub-units of 1.5 × 1.5 × 3.0 mm, which were placed using focal point displacements and shortest possible times, under thermal dose control.

**Conclusion:** We hereby present our current approach to the MRgFUS PTT on the basis of a histological reappraisal and optimized heat application to the pallidothalamic tract in the H1 field of Forel.

## Introduction

Since the advent of MRgFUS in functional neurosurgery (1), clinical experience has been gained by different groups worldwide in the fields of essential and parkinsonian tremor (2–13), obsessive compulsive disease (14), neurogenic pain (1, 15) and PD (16–18). Accuracy studies have demonstrated a mean targeting error inside the millimeter (8, 16, 19–21).

The rationale for the choice of the pallidothalamic tract over other targets in medically refractory chronic PD has been discussed in details elsewhere (16, 22, 23). Pallidothalamic fibers of the ansa lenticularis and fasciculus lenticularis (or H2 field of Forel) join 2–3 mm below the intercommissural plane and are funneled into the thalamic fasciculus (or H1 field of Forel) before reaching the thalamus. Interrupting the pallidothalamic tract at the level of H1 corresponds functionally to a pallidotomy as the majority of pallidal outputs go through it, but with a much smaller lesion size (24). Although relatively small, the pallidothalamic tract has to be sufficiently severed to produce the desired lasting clinical improvements. Optimal target volume coverage is the subject of this technical note.

## Methods and Results

### Histological Analysis

A detailed localization analysis of the pallidothalamic tract in the subthalamic area was performed on the extensive original material accumulated by Dr. Morel, partly published in different versions of the Morel's Atlas of the human thalamus and basal ganglia (25–27) and in a fiber tract study (24). Anatomical reappraisal of the interindividual variations of the pallidothalamic tract around the intercommissural plane (DV0) was conducted in order to provide maximal coverage of the tract without affecting surrounding eloquent neuroanatomical structures.

Histological sections and maps from 6 human brain hemispheres (Morel's cases Hb1-Hb5 and Hb8) cut in their axial stereotactic plane were superimposed (1) at their intercommissural plane (DV0) for distances between the center of the anterior (AC) and posterior (PC) commissures with an AC-PC distance ≥26mm, and (2) at level V0.9 mm for AC-PC distances <26 mm. This distinction is based on the observation that brains with AC-PC distances <26 mm display at level V0.9 the same histological anatomy as the one seen at DV0 in brains with larger AC-PC distances.

The AC-PC line was placed according to Morel et al. (26), i.e., the line connecting the center of both commissures as seen on the sagittal plane.

**Figure 1:**
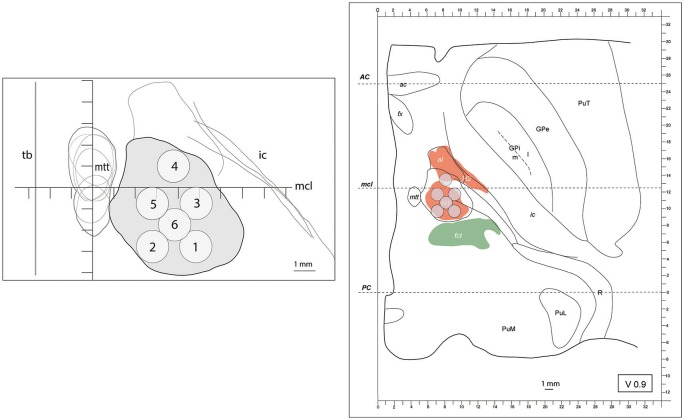
(left) outline (in gray) of the maximal extension of the PTT drawn from Myelin stains on an axial image. The target lesion sub-units (1 to 6) were adapted for intercommissural length <26mm, 0.9mm below the intercommissural plane. mtt (mammillothalamic tract), ic (internal capsule), mcl (mid-commissural line), tb (thalamic border). Coordinates of target lesion sub-unit 1: lateral 7.5mm (L 7.5), 2.7mm posterior to MCL (MCL-2.7) and 1mm ventral to the intercommissural plane (V1); 2: L5.5, MCL-2.7, V1; 3: L7.5, MCL-0.7, V1; 4: L6.5, MCL+1, V1; 5: L5.5, MCL-0.7, V1; and 6: L6.5, MCL-1.7, V1. (right) PTT target lesion sub-units projected on a modified map of the Morel's Stereotactic Atlas of the Human Thalamus and Basal Ganglia 0.9mm ventral to the intercommissural plane. For ICL ≥26mm see Figure 2.

**Figure 2:**
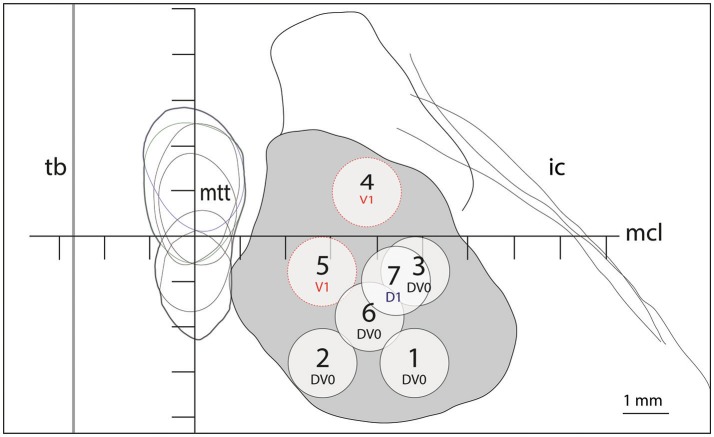
Target lesion sub-units 1 to 7 for ICL ≥26mm depicted at the level of the intercommissural plane (DV0). Target lesion sub-units 4 and 5 are projected from V1 and sub-unit 7 from 1mm dorsal (D1). Coordinates of target lesion sub-unit 1: L 7.5, MCL-2.7, DV0; 2: L5.5, MCL-2.7, DV0; 3: L7.5, MCL-0.7, DV0; 4: L6.5, MCL+1, V1; 5: L5.5, MCL-0.7, V1; 6: L6.5, MCL-1.7, DV0; and 7: L7.0, MCL-1, D1.

**Figure 3:**
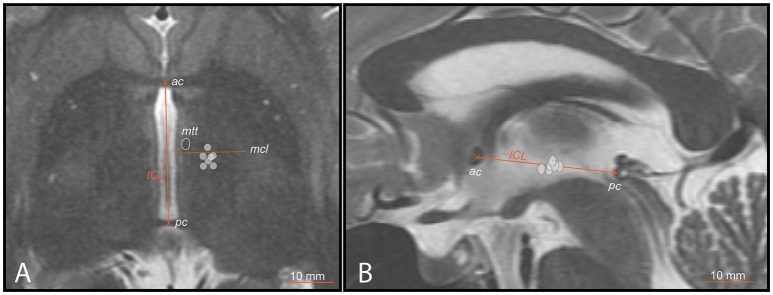
PTT target lesion sub-units (ICL ≥26mm) (gray circles) on preoperative MR images. **(A)** axial scan centered on the intercommissural plane (DV0). ICL is the distance between the center of the anterior (ac) and posterior (pc) commissures. The mid-commissural line (mcl) is represented as dotted line. **(B)** mid-sagittal scan onto which target lesion sub-units 1 to 7 (ICL ≥26mm) are projected.

Superposition was made on the mid-commissural line (MCL) [perpendicular line crossing the mid-point of the intercommissural line (ICL), see Figures 1–**3**] anteroposteriorly and on the mammillothalamic tract (MTT) in the mediolateral direction. In the Morel's atlas (26), detailed cartography was presented in patients with intercommissural distances of 25 and 26 mm. We found a mean intercommissural distance of 27.0 ± 1.6 mm measured on axial MR T2 images in our last operated 182 patients (mean age 64.1 ± 12.9 years; unpublished data). For other authors (28), the mean value of the AC-PC was even 28.3 ± 1.6 mm (*n* = 21). Cases with longer intercommissural distances (*n* = 3) were analyzed but not fully mapped in the published atlas. Their analysis did however not provide evidence for the necessity of further changes in the target location in the DV or any other direction.

At DV0 for AC-PC ≥26 mm and V1 for AC-PC <26 mm, mean distance between the center of MTT and adjacent thalamic border was 2.7 mm (SD 0.4 mm) and its mean distance to the MCL was 0.3 mm anteriorly (SD 1 mm) (*n* = 6). At D1 for AC-PC ≥26 mm and DV0 for AC-PC <26 mm, the mean distance between the center of the MTT and the adjacent thalamic border was 3.7 mm (SD 0.3 mm) and the mean position of the center of the MTT was 0.8 mm (SD 0.9 mm) anterior to MCL (*n* = 4), respectively. The MTT had on these histological samples greater variability in the anteroposterior than in the mediolateral direction, in accordance with Morel et al. (26). Its course in the subthalamic area shows a posterior to anterior and medial to lateral course toward the thalamic anterior nuclear complex.

Outlines of the pallidothalamic tract were superimposed on the intercommissural plane where it is funneled into the thalamic fasciculus (H1 field of Forel). It was identified on the basis of Myelin-stained sections and fitted onto Nissl drawn maps of the subthalamic region. The global outline of H1 in 6 human brains is represented in Figures 1, 2, including intercommissural distances between 24 and 30 mm. Target lesion sub-units of 1.5 × 1.5 × 3 mm, corresponding to the ultrasound focal region size (as given by the manufacturer) were deployed on the tract with the goal to optimize target coverage and minimize risks for adjacent structures (i.e., MTT, subthalamic nucleus, IC and somatosensory thalamus).

During treatment planning, great care was taken to respect a minimal distance of at least 2.5 mm between any target lesion sub-unit center (see Figures 1, 2) and the border of the MTT. The MTT was most of the time measurable in preoperative MR images at DV0 and V2, less often at D2. In case of insufficient preoperative image resolution, the most unfavorable position of the MTT was inferred (see cumulative outlines shown in Figures 1, 2).

Figure 1 shows on the left the outline of the maximal extension of the PTT drawn on Myelin stains of 6 human brain hemispheres partly analyzed in previous histological studies (24, 26). The target lesion sub-units (1 to 6) were adapted for intercommissural length <26 mm, 1 mm below the intercommissural plane. The right part of Figure 1 depicts the PTT target lesion sub-units projected on a modified map of the Morel's Stereotactic Atlas of the Human Thalamus and Basal Ganglia (26) 0.9 mm ventral to the intercommissural plane. Figure 2 illustrates target lesion sub-units 1 to 7 for ICL ≥26 mm depicted at the level of the intercommissural plane (DV0). Target lesion sub-units 4 and 5 are projected from V1 and sub-unit 7 from 1 mm dorsal (D1). In patients with a short dorsoventral extension of their subthalamus, target lesion sub-units 4 and 5 can be kept centered on the DV0 plane, and in rare patients with short dorsoventral subthalamus extensions as well as short intercommissural distances, all target lesion sub-units may be centered on the DV0 plane. The Figure 3 shows the planned PTT target lesion sub-units on preoperative MR images (ICL ≥26 mm).

### Procedure

Focused ultrasound procedure were performed using the ExAblate Neuro device (InSightec, Haifa, Israel) in a 3-Tesla MR imaging system (GE Discovery 750, GE Healthcare, Milwaukee, WI, USA). Procedure setup and low-power sonication rounds below 47°C to adjust the position of the thermal spot have been described previously (8, 16). Three-dimensional stereotactic coordinates of each lesion sub-unit, listed in legends to Figures 1, 2, were measured on the MR images, based on the intercommisural line and the thalamo-ventricular border. The natural transducer focal point was adjusted as close as possible (<1 mm) to the center of the PTT target, which is located 6.5 mm from the medial thalamic border (L 6.5), 1 mm posterior to the MCL (MCL-1) and on the intercommissural plane (correspondingly 1 mm ventral to the intercommissural plane in ICL <26 mm). As depicted in Figures 1, 2, final temperature sonications were applied to the different target lesion sub-units according to their pre-planned focal point coordinates (1 to 6 in Figure 1 and 1 to 7 in Figure 2). Each sonication had the shortest possible time and the corresponding power in order to provide a thermal dose of 240 CEM at each focal point which represents a conservative value corresponding to a 100% probability of lesion (29–31) in a volume of 1.5 × 1.5 × 3.0 mm. According to thermal doses achieved, adaptations of the application of sub-targets 6 or 7 were sometimes deemed necessary, one or two target sub-units being abandoned or the position of some of them being individually adapted.

Temperature monitoring was also performed in the MTT during each sonication, great care being taken never to reach temperatures over 43°C in it. In addition, all thermal doses for the MTT were calculated and their sum was kept below 2 CEM. Depending on temperature monitoring in the MTT in previous sonications, target lesion sub-unit 5 was sometimes reprogrammed 0.5 mm laterally or even left aside.

Intraoperative MR T2 examinations were performed at the end of the intervention as early as possible after the last sonication, with the body coil and with the patient in treatment position. Postoperative MR examinations with the 32-channel head coil were performed 2 days after the intervention.

Corticosteroids were routinely applied in all patients treated with this protocol, intravenously within 1 h following last sonication (usually 40 mg Solumedrol) and after 12 h. Dexamethasone (3 × 2 mg per day) was given p.o. the following 3-4 days.

**Figure 4:**
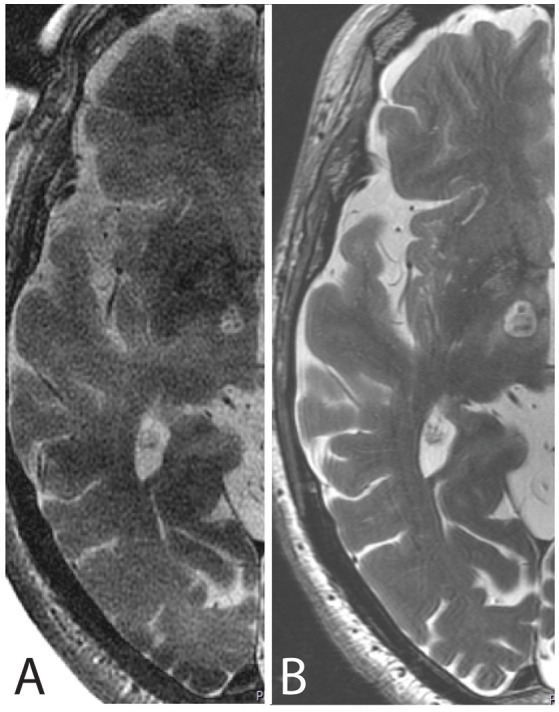
MR axial scans cut through the intercommissural plane after PTT taken **(A)** intraoperatively with the body coil and **(B)** 48 h after the procedure with the 32-channel head coil.

Figure 4 depicts one example of the final lesion site after completing the last target lesion sub-unit intraoperatively and 48 h after the procedure on MR T2 axial scans.

All patients treated with this protocol signed an informed consent form after having been fully informed about the treatment, its results and risks. No ethical approval was sought because MRgFUS PTT is approved by the swiss health state department and covered by swiss social insurances.

## Discussion

Our experience of lesioning the pallidothalamic tract on its way to the thalamus has led us, after re-analysis of recurrences or partial symptom control, to develop a targeting protocol aiming at better spatial coverage of the target. It takes into account interindividual anatomical variability and adopts the strategy of applying small thermolesions with shortest possible sonication times, under thermal dose control and moving the focal point of the MRgFUS system. Neither repetition of high temperature (54–60°C) sonications nor prolonging sonication time without moving the focal point had brought sufficient consistency in patient symptom control. The application of the complete series of sub-targets proposed is not always possible, requiring strategy adaptations. Immediate tissue changes due to the thermolesion, mainly intra- and perilesional edema and increase in local blood flow can reduce the efficacy of neighboring sonications. This situation can be exacerbated by a low skull density ratio and preexisting tissue changes, mainly microangiopathic. In this context, it is obvious that the time spent between each sub-target lesion application should be as short as possible.

We currently still cannot rely on diffusion MR tractography of the pallidothalamic tract for lesioning purposes due to relatively poor radiological accuracy (32) and particularly when deterministic based methods are used (33). In this context, we still favor an atlas-based approach taking interindividual variability into account, crosschecking the planned coordinates with high resolution MR images in stereotactic planes for relevant adjacent visible structures. For future developments, motion-corrected ultra-high resolution MR methods have been shown to permit very high quality isotropic images with nominal resolution of up to 350 μm (34) or even 250 μm (35, 36). These might permit a direct visualization of the PTT in dedicated preoperative MR scan, which could reduce the target volume to be covered for individual patients.

## Conclusion

This study presents our current approach to the MRgFUS pallidothalamic lesioning which was developed because the two strategies of application repetitions and increase of application time failed to prevent symptom recurrences. It takes into account interindividual variability, and is characterized by the applications under thermal dose control of multiple small thermal lesions using shortest possible times, and distributed in a preplanned manner to optimize target coverage.

## Author Contributions

MG, DM, and DJ contributed to the conception and design of the study and acquisition, analysis, and interpretation of the data and co-drafted the manuscript. CF co-drafted the manuscript. All authors read and approved the final manuscript.

### Conflict of Interest Statement

MG, DM, and DJ were employed by company SoniModul Ltd., Center of Ultrasound Functional Neurosurgery, Solothurn, Switzerland. The remaining author declares that the research was conducted in the absence of any commercial or financial relationships that could be construed as a potential conflict of interest.

## Abbreviations

ICL: intercommissural line
MTT: mammillothalamic tract
IC: internal capsule
CEM: cumulative equivalent minutes at 43°C
MCL: mid-commissural line
MRgFUS: MR-guided high intensity focused ultrasound
PTT: pallidothalamic tractotomy
PD: Parkinson's disease.
